# Research into the Failure Mechanism and Reliability of an Active Frequency-Selective Surface in Complex Environments

**DOI:** 10.3390/ma18061354

**Published:** 2025-03-19

**Authors:** Zheng Wei, Bin Suo, Chunping Zhou, Yueqi Li, Feng Zhang

**Affiliations:** 1 Key Lab for Airborne Hi-Performance Electro-Magnetic Window, AVIC Research Institute for Special Structures of Aeronautical Composites, Ji’nan 250102, China; danielwei1994@163.com (Z.W.); zcp99241@126.com (C.Z.); 15105410361@163.com (Y.L.); 2 School of Information Engineering, Southwest University of Science and Technology, Mianyang 621010, China; 3 Department of Engineering Mechanics, Northwestern Polytechnical University, Xi’an 710072, China; nwpuwindy@nwpu.edu.cn; 4 National Key Laboratory of Strength and Structural Integrity, Xi’an 710072, China

**Keywords:** active electromagnetic functional structure, active frequency-selective surface (AFSS), failure modes and mechanisms, fault excitation test

## Abstract

This paper presents research into the failure modes and mechanisms of active electromagnetic functional structures. An active electromagnetic functional structure is usually achieved by loading active devices on a frequency-selective surface and designing an active frequency-selective surface (AFSS) structure with adjustable transmission and cutoff characteristics through feeding. This has shown significant advantages in electromagnetic countermeasures for fighter jets and has become one of the key technologies for the future development of such equipment in the context of integration and intelligence. The active electromagnetic functional structure is different from the traditional composite material structure because it includes active functional devices to realize the electromagnetic function but also includes a structural component to bear the complex environment. The failure modes are also more complex, including both structural failure and functional degradation or even failure caused by device failure, and the coupling relationship between different failure modes is complex. However, the failure modes of active electromagnetic functional structures in complex environments have received little research attention, and the corresponding fault modes and mechanisms are poorly understood, thereby limiting their practical applications. Thus, in the current study, environmental stress analysis is performed to verify the main failure modes and mechanisms of active electromagnetic functional structures through fault excitation tests. Importantly, the obtained results clarify the stress sensitivity of the structure and provide effective support for its engineering applications.

## 1. Introduction

The concept of the active frequency-selective surface (AFSS) was initially proposed by Chang et al. [1], who loaded a PIN diode between the square ring and the dipole and switched the filtering characteristics by controlling the bias voltage of the diode. Subsequently, Ekmekci et al. [2] designed a multiband tunable structure based on a microsplit open-ring resonator, and adopted a microelectromechanical system (MEMS) to control the resonator split state and achieve multiband frequency tunability. In recent years, the AFSS structure has gradually received growing research interest, and its functions have become more diversified [3]. For example, Ghosh et al. [4] designed a ring frequency-selective surface (FSS) loaded with a PIN diode to realize absorption/reflection by controlling the switch of the diode. Subsequently, the same group [5] designed an AFSS structure combining a square ring and four metal branches to realize absorption/reflection at 2.38 GHz. Later, they expanded this to a 3.56–8.16 GHz wideband absorption/reflection function through structural improvements [6]. Chen et al. [7] designed a switched beam antenna based on an active frequency-selective surface (AFSS), achieving high gain and 360-degree coverage with highly directional characteristics. Shah et al. [8] designed an active frequency-selective surface with dual-band reconfigurability, exhibiting high angular stability, low insertion loss, and dual-band passband/stopband tunability.

Consequently, the AFSS structure has gradually become an important research direction. In China, this area has developed rapidly under the demand of practical application, with various universities and national defense units being engaged in relevant theoretical research. For example, Yanping et al. [9] designed a metal tree-shaped wave absorption structure, which achieved an almost 100% perfect wave absorption. In another study, Xu et al. [10] designed a structure based on a square resonant ring and a cross-Jerusalem structure, which achieved an absorption effect of ≥97% at three frequency points. Furthermore, Pengcheng et al. [11] designed a unit structure with two open resonant rings connected by a resistor, achieving a wave absorption rate of >90% in the 8.1–11.9 GHz frequency band. In the last decade, certain academic achievements have also been reported. For instance, Kai et al. [12] designed a flexible wideband tunable microwave structure, while Shaobo et al. [13,14] reported a temperature-controlled adjustable structure, and Xiaoyu et al. [15] produced a multi-functional active structure with switch-controllable and frequency-adjustable functions. Junxie [16] proposed a light-controlled method to replace the commonly used electrical control structure in AFSS. Wu et al. [17] designed a composite AFSS structure, achieving integrated properties of wave absorption and transmission. Yannan et al. [18] introduced a novel ultra-wideband absorber unit model operating in the S/C band. Ruizhen et al. [19] designed a square symmetric metamaterial terahertz multi-frequency absorber capable of addressing issues related to high-frequency, multi-frequency, and high absorption rate performance. Zimeng et al. [20] developed an electrically controlled angular reflector based on a single-sided AFSS, achieving real-time control of wide-angle domains using passive scatterers.

Despite such progress, product maturity and the reliability of their corresponding practical applications are low. Considering the wide application of the AFSS structure in radar radomes, weapons equipment, aircraft, and other military fields, the environmental stresses associated with these complex environments can easily lead to the failure of parts or solder joints, ultimately affecting the material performance. Therefore, an in-depth study into the failure mechanism of the AFSS structure would be expected to help to determine the causes of failure, identify deficiencies in the design and process methods, and provide a scientific basis for improvement measures. In addition, a reliability assessment could identify the weak links of the AFSS structure, propose targeted improvement plans, and determine whether the product meets the reliability requirements, which is particularly crucial in the context of military applications.

Since the AFSS structure differs from those of general electronic products, it is necessary to carry out special research to explore its failure modes and mechanisms under the effects of flight vibrations, humidity, heat, and other environmental stresses. This can be achieved through fault mode impact and hazard analysis (FMEA), fault excitation tests, and failure analysis (FA), among other methods [21,22]. In addition, introduced during the late 1980s and early 1990s, highly accelerated life testing (HALT) and reliability enhancement testing (RET) are established reliability testing techniques. They are designed to swiftly uncover defects, playing a crucial role in the evaluation and improvement of product reliability [23,24]. More specifically, HALT technology has been widely used in the electronics, automobile, rail transit, medical treatment, communications, and aerospace fields [25]. Yan et al. [26] A Study on the Application of Highly Accelerated Life Testing (HALT) to Body Controllers: HALT simulates a wide array of stress conditions that a product may face across its entire lifecycle by imposing stresses significantly exceeding normal operational limits. This method allows for the preemptive identification of potential failure modes. Sun [27] focused on a specific typical surface-mounted electronic component as the object of study, leveraging sophisticated computer simulation technology to uncover its vibration fatigue failure mechanisms under accelerated test conditions. Weichuan et al. [28] performed HALT testing on digital instrument control equipment, addressing potential failure modes that could not be avoided during the design phase. Similarly, RET technology was employed in the radio proximity fuze design of Sidewinder missiles to improve product reliability through an enhanced design, enhanced stress screening, and screening verification methods [29]. Jie et al. [30] applied RET technology to the design improvement of a military DC power supply, addressing design flaws. Cao et al. [31] based on a certain type of solenoid valve used in the braking system of rail transit, a workflow for its reliability enhancement test was designed, test profiles for each stage were formulated, and corresponding tests were conducted. Moreover, compared with single-stress tests, multi-stress combination tests are more effective in stimulating product defects [32,33,34,35,36].

However, current research into the AFSS structure mainly focuses on the design and the realization of target functions, with the systematic analysis of its failure mode and failure mechanism having received little attention. Considering the practical application requirements of the AFSS structure, it is necessary to perform failure mechanism analysis to improve product reliability and ensure adaptation to complex environments.

Thus, in the current study, the AFSS structure is subjected to FMEA to identify its key failure modes, while fault excitation tests are performed to verify the weak structural links and provide a scientific basis for product design and performance optimization. Considering the necessity to determine the best means of employing the fault excitation data to quantify its ability to evaluate reliability improvements, this study focuses on the failure mechanism and reliability evaluation of the AFSS structure. It is expected that this work will provide theoretical support and practical guidance for subsequent reliability design and engineering applications through systematic tests and analyses. The architecture of the paper is organized as follows: Section 2: Materials and Methods, which describes the basic AFSS structure and test process; Section 3: Results and Discussion, which analyzes the test results and the typical failure modes, and test conclusions are obtained.

## 2. Materials and Methods

### 2.1. The Basic AFSS Structure

**Figure 1:**
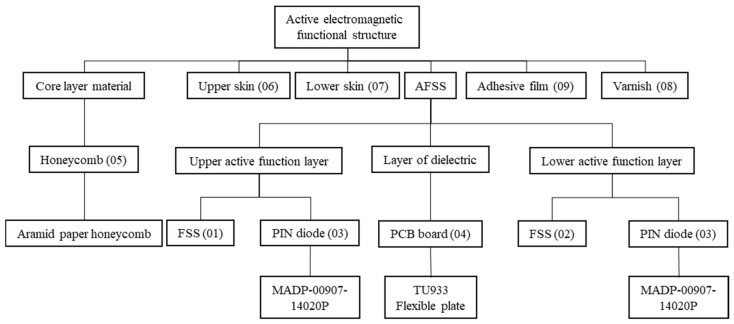
Structural diagram outlining the typical structure of a source device.

The typical structure of the active electromagnetic functional structure is composed of the AFSS, upper skin, lower skin, honeycomb, varnish, and film. These components can be regarded as a multilayered material structure. A structure diagram outlining the typical structure of a source device is presented in Figure 1.

**Figure 2:**
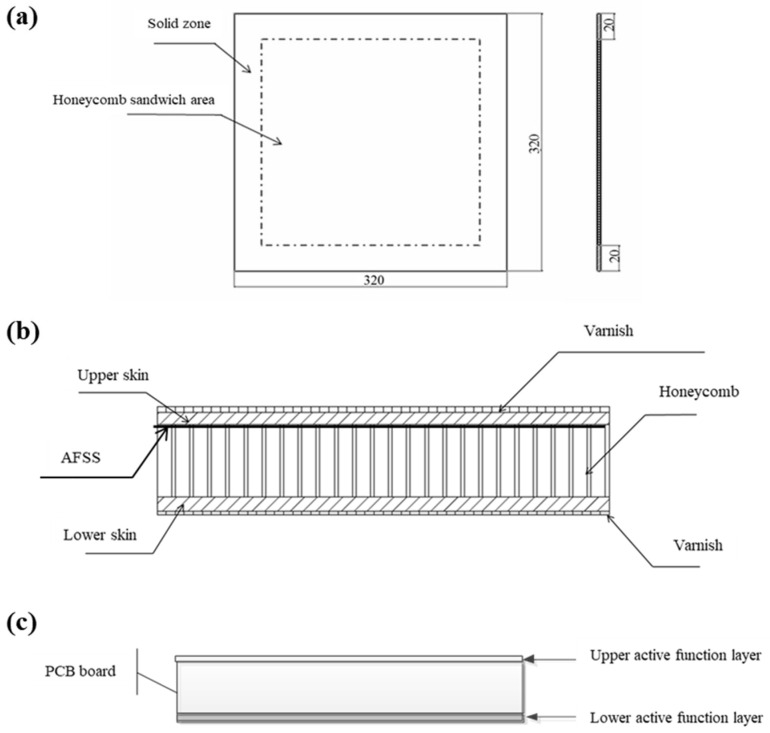
Schematic diagram showing the typical structure of the source device. (**a**) Front view, (**b**) side view, and (**c**) the PCBA.

In this study, the test object was a typically structured source device measuring 320 mm × 320 mm, as shown in Figure 2a. The honeycomb sandwich area in the middle of this structure measures 280 mm × 280 mm, as can be clearly seen in the side-view image presented in Figure 2b. The outer area, with a width of 20 mm, is a solid area consisting of a full-skin structure. The middle printed circuit board assembly (PCBA) constitutes the core functional component, as shown in Figure 2c. From top to bottom, the first layer is the component mounting layer, the second layer is the printed circuit board (PCB, Rogers 5880 material, MADP-000907-14020P) with a thickness of 0.127 mm, and the third layer is the component mounting layer.

### 2.2. Test Sample and Test System Design

**Figure 3:**
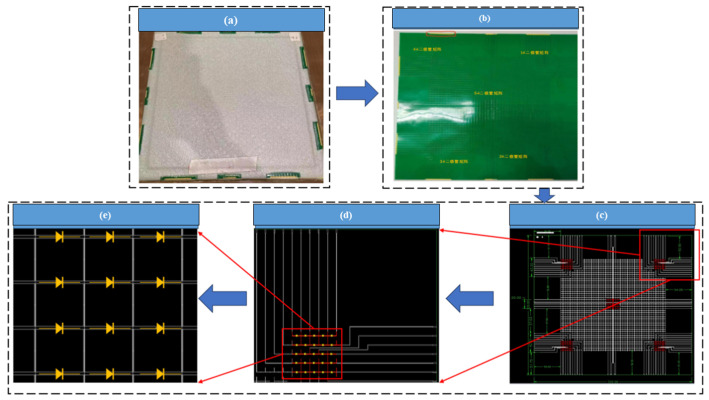
AFSS structure diagram. (**a**) The test piece, (**b**) the PCBA, (**c**) the specially designed AFSS, (**d**) a single 5 × 5 diode array, and (**e**) the principle of the test system.

A photographic image of the AFSS test piece and the principle of the test system are provided in Figure 3. Each sample consists of a PCBA, a skin layer, and a honeycomb structure, as shown in Figure 3a. The design test board is used to judge the abnormal state of the diode, and it is required to detect the three states of the diode, namely, normal, open circuit, and short circuit. To be able to locate faults in the diode array during the test, the sample employed for the fault excitation test was specially designed, as shown in Figure 3c. More specifically, the upper and lower surfaces of the tested circuit consisted of five 5 × 5 diode arrays, numbered 1 to 5. The developed test circuit can test a single 5 × 5 diode array, and so a total of five test circuit boards are required. This design is convenient for fault location during the testing process, in addition to the identification of open circuit failure. Figure 3d,e show the locally enlarged structures of the test system.

### 2.3. Design and Implementation of the AFSS Structure Fault Excitation Test

#### 2.3.1. Design of the Test Scheme

**Figure 4:**
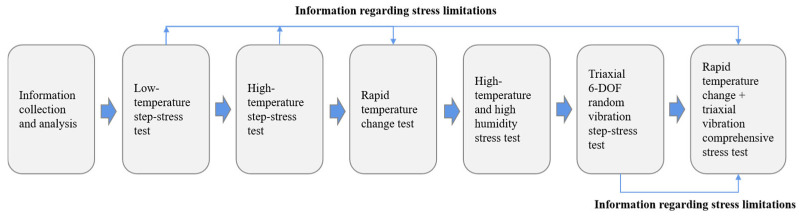
Flow diagram representing the typical structure fault excitation test of the source device.

Considering that the fault mode of the active electromagnetic structure is complex and the fault mechanism remains unclear, fault mode impact and hazard analysis is performed on the typical active electromagnetic structure in a typical service environment to design a fault excitation test under high accelerated stress conditions. In addition, multi-level and cross-scale fault mechanism detection and analysis are performed on the test parts after the test. Furthermore, the main failure mode and failure mechanism are clearly defined to support the reliability analysis and product application of the active electromagnetic function structure. More specifically, the overall process employed to perform the fault excitation test for typical source device structures is outlined in Figure 4.

During the test, the typical structure of the source device is mainly tested with regards to its appearance and the diode matrix text. More specifically, a visual inspection and a magnifying glass (5×) are used to observe cracks, bulges, peeling, and other phenomena on the sample surface. In addition, for the diode matrix test, a test board is used to assess the state of the diode and detect whether there is a short circuit, open circuit, or other faults.

#### 2.3.2. Test Process

The temperature data for the typical source device structure are listed in Table 1. According to these details, the storage temperature range of the typical source device structure is the same as its operating temperature range, namely, −55–125 °C.

**Figure 5:**
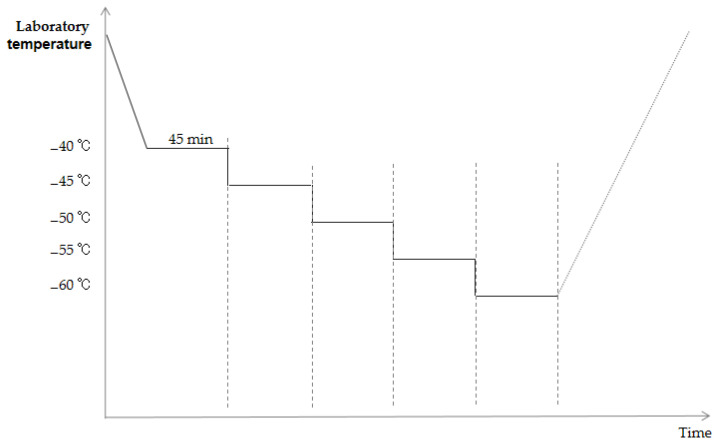
Profile of the low-temperature step-stress test.

For sub-test 1, which involves the low-temperature step-stress test, the temperature of the initial stress condition was set to −40 °C. The profile of the low-temperature step-stress test is shown in Figure 5. More specifically, starting at −40 °C, the temperature was reduced in steps of −5 °C, wherein each temperature was maintained for 45 min (30 min thermal balance, 15 min hold). Since the low-temperature operation and storage limit of the diode are −55 °C, the test cutoff temperature was set to −60 °C, and the relative nominal value was assessed to determine whether a margin existed. A 5 V power supply was used throughout the test, and the state of the diode was scanned after each cycle.

**Figure 6:**
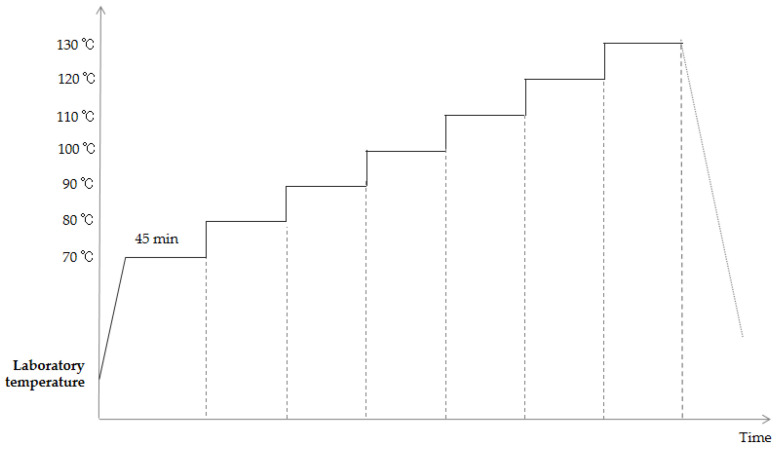
Profile of the high-temperature step-stress test.

For sub-test 2, which represents the high-temperature step-stress test, it should be noted that the minimum high temperature limit of the typical structural components of a source device is 125 °C. It can be seen from the analysis that the maximum working environment temperature of the typical source device structure is ~72 °C. After comprehensive consideration, the starting high temperature for the test was defined as 70 °C, and a step size of 10 °C was employed, as shown in Figure 6. Each step was maintained for 45 min (30 min heat balance time, 15 min hold). According to the stress survey of the tested product in Table 1, the cutoff temperature was set at 130 °C. As above, a 5 V power supply was employed throughout the test, and the state of the diode was scanned after each cycle.

**Figure 7:**
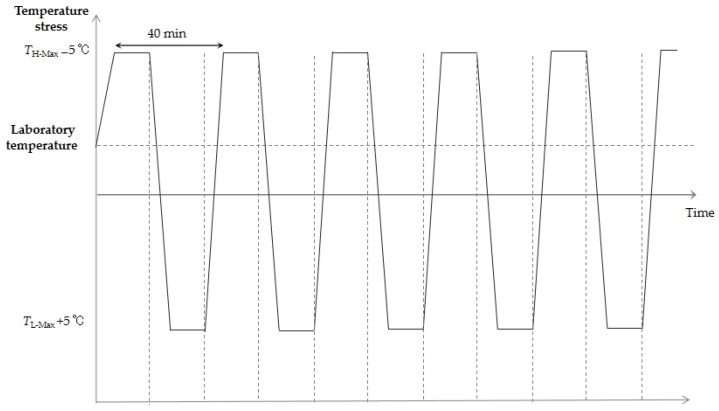
Profile of the rapid temperature change test.

For sub-test 3, namely, the rapid temperature change test, the working limit stress of the power supply board obtained from the low-temperature step test was defined as *T*_L-Max_, while the working limit stress obtained from the high-temperature step test was defined as *T*_H-Max_. The test profile for the rapid temperature change stress is outlined in Figure 7. More specifically, the temperature varied between the low temperature *T*_L-Max_ + 5 °C and the high temperature *T*_H-Max_ − 5 °C. Each low- and high-temperature step was maintained for 15 min, the temperature variation rate was 40 °C/min, and each cycle had a duration of 40 min. A total of 5 cycles were performed, a 5 V power supply was employed throughout the test, and the state of the diode was scanned after each cycle.

**Figure 8:**
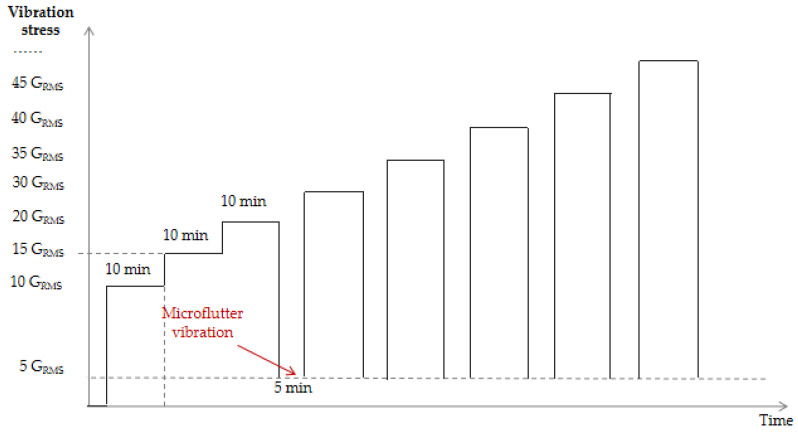
Profile of the triaxial 6-DOF random vibration step-stress test.

According to the stress survey performed on typical aircraft, the minimum root-mean-square acceleration of the random vibration test for a typical source device structure is 9.995 G_RMS_ (vertical), while its maximum value is 11.229 G_RMS_ (lateral). Therefore, the initial stress employed for sub-test 4, which is the triaxial 6-DOF random vibration step-stress test, was set at 10 G_RMS_. The test profile for this test is presented in Figure 8. More specifically, using an initial stress of 10 G_RMS_, a step size of 5 G_RMS_ was employed, and each step was maintained for 10 min. Beyond 20 G_RMS_, a 5 min microtremor vibration stage was added for each step, wherein the stress was allowed to return to 5 G_RMS_, held for 5 min, and subsequently stepped up to the next stress level. As above, a 5 V power supply was used throughout, and the state of the diode was scanned once for each step.

**Figure 9:**
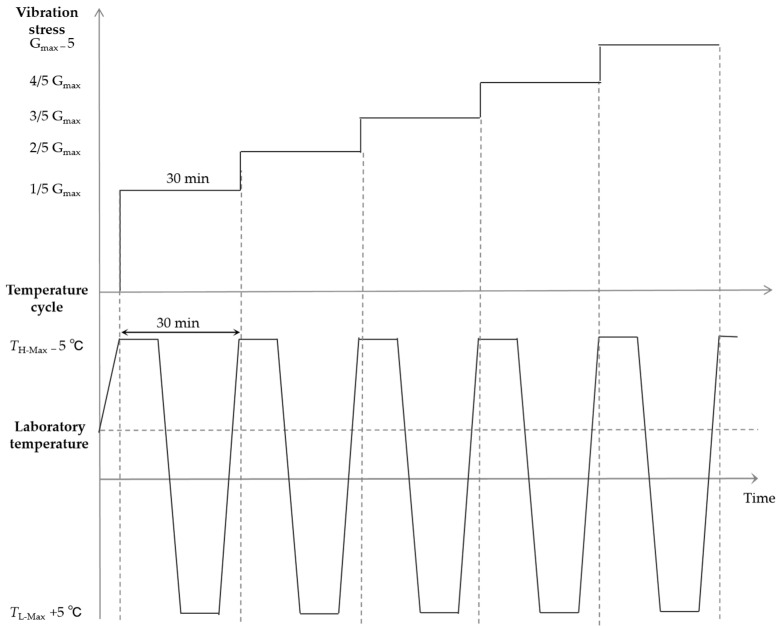
Profile of the rapid temperature change + triaxial vibration comprehensive stress.

To stimulate the fault of the typical source device structure under extreme environmental conditions, the rapid temperature change + triaxial vibration comprehensive stress was employed (namely, sub-test 5). For this purpose, the working limit stress obtained from the low-temperature step test was set as *T*_L-Max_, the working limit stress obtained from the high-temperature step test was set as *T*_H-Max_, and the vibration limit stress obtained from sub-test 4 was set as G_max_. As presented in Figure 9, sub-test 5 was performed as follows. Firstly, temperature cycling was performed from the low temperature *T*_L-Max_ + 5 °C and the high temperature *T*_H-Max_ − 5 °C. Each cycle had a duration of 30 min, the temperature variation was 40 °C/min, and a total of 5 cycles were employed. For the vibration, the starting stress was set as 1/5 G_max_, the step size was 1/5 G, and the cutoff stress was G_max_—5 G_RMS_. Each step corresponds to a single temperature cycle. As above, a 5 V power supply was employed throughout the test, and the state of the diode was scanned after each cycle.

For sub-test 6, which constitutes the high-temperature and high-humidity stress test, the working limit stress obtained from the high-temperature step test was set as *T*_H-Max_. Notably, since relative humidity (RH) >90% should be maintained in the wet heat test chamber, the maximum temperature can only reach 90 °C. Therefore, when *T*_H-Max_ ≤ 85 °C, the high-temperature and high-humidity test was carried out at a temperature of *T*_H-Max_ and a humidity of 90%RH. Otherwise, a temperature of 90 °C and a humidity of 90%RH were used to provide the appropriate humid thermal stress conditions. A 24 h cycle time was employed for a total of 3 cycles (i.e., 72 h test). A 5 V power supply was used, and the state of the diode was scanned after each cycle.

## 3. Results and Discussion

### 3.1. Test Results

Fault excitation tests were performed for the AFSS structure using different kinds of stress, and the results are summarized in Table 2. It can be seen that the typical source device structure does not fail under low-temperature and humid thermal stress conditions. However, at high temperatures, open-circuit and short-circuit failure began to take place beyond 230 °C; however, it should be noted that this temperature far exceeds the temperature of actual product usage. In addition, it was deduced that the main diode failure mode is mainly open-circuit failure, although at extremely high temperatures (270 °C), a large number of short circuits were detected. Furthermore, short-circuit failure also occurred under the rapid temperature change + triaxial vibration comprehensive stress test conditions.

In addition, the new failure rate of the diode under the various test profile conditions was measured, and the results are shown in Table 3.

### 3.2. Analysis of the AFSS Structure Failure Mode and Mechanism

Failure analysis was subsequently performed for the AFSS structure subjected to the fault excitation tests. Specific details regarding these tests and their requirements are outlined in Table 4.

Failure analysis was subsequently performed for six typical source device structures subjected to the fault excitation tests. Detection and slice analyses were carried out for the board level and the diode, while the failure mode and failure mechanism of the PIN diode, solder joint, PCB, and multilayer structure were analyzed in detail. As a result, 15 typical failure modes were identified, and the failure mechanisms were analyzed, as summarized in Table 5.

## 4. Conclusions

In this study, environmental stress analysis was performed to verify the main failure modes and mechanisms of active electromagnetic functional structures through fault excitation tests. Upon combination of the fault excitation test data and the failure analysis results, the reliability level of the typical source device structure was evaluated. It was found that the reliability level of this structure was excellent under low-temperature, high-temperature, vibration, and humid environments. No new open-circuit or short-circuit failure occurred in the low-temperature step, high-temperature step (70–130 °C), or humid heat tests. For the vibration step test, the proportion of open-circuit failure was 1.56% at 50 G_rms_, while in the rapid temperature change test, the proportion of open-circuit failure was 1.92%, and no short-circuit failure was observed. In addition, the triaxial 6-DOF random vibration step-stress test demonstrated 2.22% open-circuit failure and 0.27% short-circuit failure. In the high-temperature step test (140–270 °C), the proportion of open-circuit failure reached 28.1%, while the proportion of short-circuit failure was 2.72%, both of which were observed mainly beyond 210 °C. However, from the failure analysis results, it was deduced that the main cause of failure was not failure of the PIN diode itself but an excess temperature that exceeded the melting point of the solder, ultimately resulting in the solder joint breakage and a resulting short circuit. The typical source device structure was also found to exhibit a small degree of breakage in the initial state, which was attributed to virtual welding and PIN diode cracking. During the SMT (refers to the entire manufacturing process of surface-mounted technology) process, significant defects exist, including the mismatch between the diode and the pad, in addition to deflections, voids, and virtual welding. These results therefore suggest the necessity to further optimize the SMT process.

## Figures and Tables

**Table 1:** Temperature information for the typical structural components of the source device.

Serial Number	Device/ Material Name	Model/Brand Number	Storage Temperature Range	Operating Temperature Range
1	Diode	MADP-000907-14020P	−55–125 °C	−55–125 °C
2	PCB board	Rogers 5880	≤150 °C	≤150 °C
3	Solder	Sn_63_Pb_37_ solder	<183 °C	<183 °C
4	Honeycomb	Aramid paper honeycomb	<155 °C	<155 °C
5	Skin	A glass fiber reinforced composite	<155 °C	<155 °C
6	Adhesive film	-	<155 °C	<155 °C
7	Varnish	Epoxy polyamide	<155 °C	<155 °C

**Table 2:** Summary of the fault excitation test results.

Serial Number	Excitation Stress	Maximum Stress	Open-Circuit Failure Condition	Short-Circuit Failure Condition
1	Low-temperature step test	−70 °C	None	None
2	High-temperature step test	High temperature step 1 (Max. 130 °C)	None	None
		High temperature step 2 (Max. 270 °C)	At 230 °C, additional circuit failure occurs, and the number of circuit failures increases gradually with increasing temperature.	Short-circuit failure occurs at 270 °C
3	Rapid temperature change test	60– 130 °C	Three new break failures	None
4	Triaxial 6-DOF random vibration step stress test	50 G_RMS_	Three new open-circuit failures, all occurring at matrix bit #5	None
5	Rapid temperature change + triaxial vibration comprehensive stress	130 °C, 50 G	Five new circuit breaker failures, all occurring in E_z_5	One short-circuit failure
6	High-temperature and high-humidity stress test	90 °C, 100%RH	None	None

**Table 3:** Summary of the failure rates of the different test sections in the fault excitation tests.

Serial Number	Diode New Failure Rate	
	Open Circuit	Short Circuit
1	0%	0%
2 (step 1)	0%	0%
2 (step 2)	28.10%	2.72%
3	1.92%	0%
4	1.56%	0%
5	2.22%	0.27%
6	0%	0%

**Table 4:** List of the equipment required for each test process.

Serial Number	Test Object	Test Items	Test Equipment	Purpose of Testing
1	Whole board	Visual inspection of appearance	Magnifying glass, stereomicroscope	Appearance of defects
2		X-ray detection	X-ray system	Observation of anomalies in the wiring
3		C-SAM	Ultrasonic scanning microscope	Presence of voids, layering, etc., in the multilayer structure
4	Diode	Visual inspection	Stereomicroscope	Appearance of defects
5		C-SAM	Ultrasonic scanning microscope	Scanning electron microscopy observations
6		SEM and EDS analysis	Scanning electron microscope, energy dispersive X-ray spectroscopy instrument	Micromorphology observations, elemental composition analysis
7		Slice analysis	Stereomicroscope, metallographic microscope, scanning electron microscope, energy dispersive X-ray spectroscopy instrument	Observation of the diode internal morphology, defect detection
8	Slice analysis	Solder joint analysis	Stereomicroscope, metallographic microscope, scanning electron microscope, energy dispersive X-ray spectroscopy instrument	Solder joint defect detection, IMC layer detection, analysis of harmful components
9		Line analysis	Stereomicroscope, metallographic microscope, scanning electron microscope, energy dispersive X-ray spectroscopy instrument	Circuit defect detection, analysis of harmful components
10		Pad analysis	Stereomicroscope, metallographic microscope, scanning electron microscope, energy dispersive X-ray spectroscopy instrument	Analysis of the thickness and morphology of the pad coating
11		Multilayer structure	Stereomicroscope	Observation of defects, such as delamination and voids

**Table 5:** Summary of the main failure modes and failure mechanisms of the typical source device structures.

Failure Mode Number	Part	Failure Mode	Failure Mechanism	Test Results
1	PIN diode	Pole rupture resulting in diode breakdown and short-circuit failure	During vibration or thermal fatigue, pole rupture and breakdown take place, resulting in the formation of a short circuit.	Not found.
2		Inter-electrode solder melt adhesion and short-circuit failure	When the high temperature exceeds the melting point, the solder at both ends of the PIN diode dissolves, and the dissolution process leads to connection of the electrodes at the two ends, resulting in short-circuit failure.	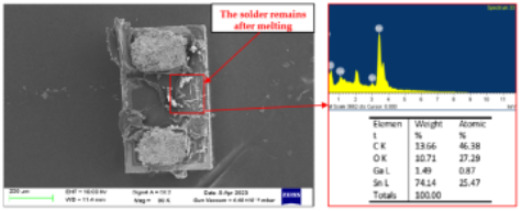
3		The electrode is broken at the bond of the tube core, and the circuit fails.	Under the action of vibration, thermal stress, etc., the bond between the electrode and the tube core may break, resulting in open-circuit failure of the PIN diode.	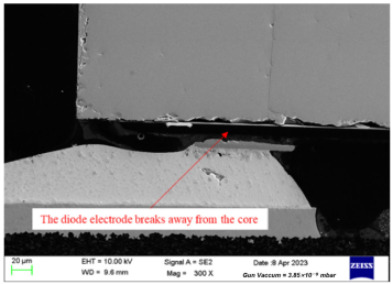
4		Body cracking and open-circuit failure	Random vibrations, temperature cycling, and high temperatures cause cracking of the PIN diode body.	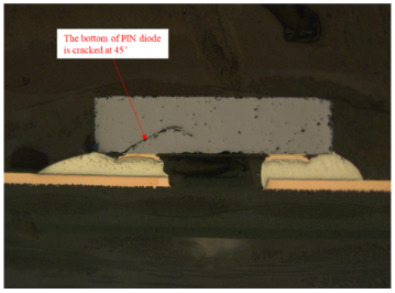
5	Solder joint	Solder joint cavities	(1) PCB Test Voids: These are caused by impurities on the PCB solder pad surface, oxidation films on the pads, or residual unexpelled flux volatiles during the assembly process. (2) Internal Cavities: Such cavities typically form during the reflow soldering process when molten solder retains the volatiles from the flux during curing. These cavities, being unaffected by external factors, are usually small in size.	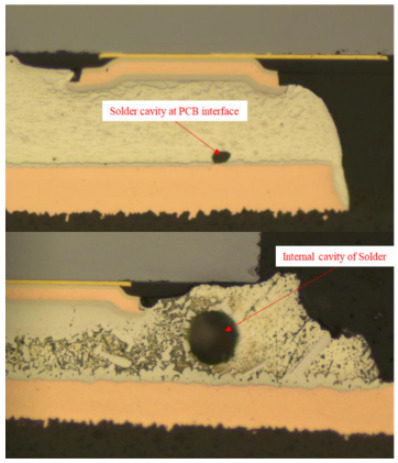
6		Solder joint cracking	(1) Thermal stress leads to fatigue cracking: (1) The coefficient of thermal expansion between the different materials is not matched, and the thermal mismatch caused by long-term thermal stress during the service process leads to volume contraction/expansion of the component under the temperature load. Stress alternations easily form at the joint and accumulate damage. (2) From a macroscopic viewpoint, the thermal load will cause the solder at the boundary of the contact interface to produce high stress, while the thermal cycle load will promote the continuous generation of alternating stress in these areas, leading to crack initiation. Finally, the crack expands until the complete interface is delaminated or solder joint fracture occurs. From a microscopic viewpoint, the grains are gradually coarsened, the gap between the grains leads to the formation of grain boundary holes, and the continuous increase in the number of holes leads to crack initiation and expansion. (2) Mechanical stress leads to cracking: Under the long-term random load, the solder joint will appear elastic and undergo plastic deformation, resulting in the rapid accumulation of fatigue damage, and eventually leading to cracking of the solder joint. (3) Poor growth of the intermetallic compounds (IMCs) results in cracking: The IMCs are two or more metal components that differ from the components present in the ordered crystal structure of the main structure. The formation and quality of the IMC impact the mechanical and electrical performances of the solder joint. The IMC is generally both hard and brittle, and a thicker IMC layer tends to have a more significant detrimental effect on the solder joint strength. An IMC layer thickness of 1–3 μm is considered optimal, <5 μm is considered normal, and >5 μm will lead to reduced solder joint tensile strength and fatigue life, in addition to facile cracking under thermal stress and mechanical stress.	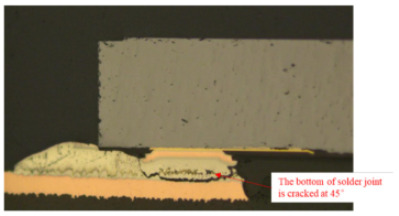
				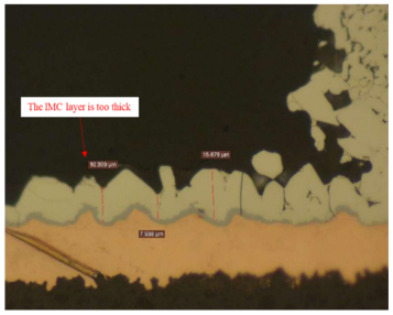
		Solder spot virtual welding	(1) The PIN diode is skewed and not aligned with the pad. (2) The solder joint position is contaminated by oxides, making it difficult to solder.	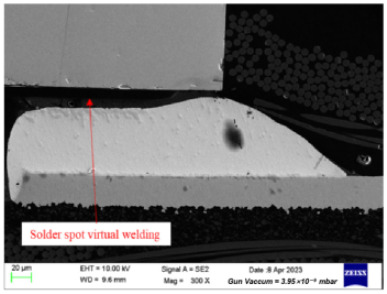
7		Inadequate solder melting	At high temperatures, secondary melting occurred, and an insufficient temperature led to a sword-like solder, coarse grains, and other phenomena. At this time, the solder joint strength is low, and it can easily crack or even fracture under the action of thermal and mechanical stresses.	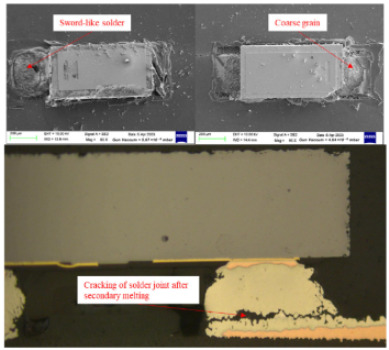
8	PCB	PCB substrate cracking	(1) The CTE (coefficient of thermal expansion) of the PCB substrate is relatively high. During the lead-free reflow soldering process, the mismatch in the expansion coefficients between the resin in the heating section and the metal copper foil leads to inconsistent thermal expansion strides in the PCB. The CTE describes the rate of change in length or volume per unit of a material in response to a change in temperature. (2) During high-temperature stepping and warm cycling, the thermal expansion coefficient of the PCB board and skin differed from one another, and the process of skin deformation produced a greater tensile stress on the PCB substrate resin material.	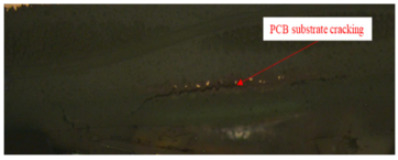
9		Mask (green oil) peeling, cracking, and detachment	(1) A small amount of water vapor is present inside the PCB board, and the copper surface is oxidized at high temperatures, thereby reducing the binding force between the green oil and the copper surface. (2) Under continuous high-temperature conditions, the green oil, the PCB substrate, and the copper layer undergo large degrees of thermal deformation.	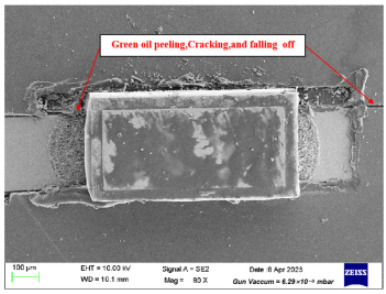
10		Line oxidation	At high temperatures, the skin becomes unstuck, and a large area of discoloration was observed for the green oil, in addition to cracking and other phenomena. The protective effect of the line is greatly weakened, rendering the copper susceptible to oxidation at high temperatures.	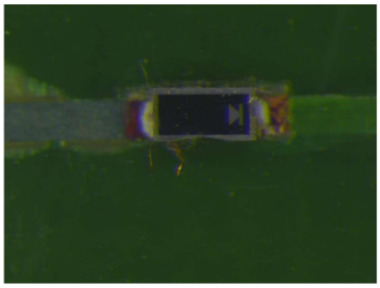
11		Uneven and insufficient pad thickness	(1) The chemical etching process does not maintain uniformity. (2) The consistency of copper thickness does not meet the requirements for thickness uniformity, solder mask integrity, component placement, and thermal management.	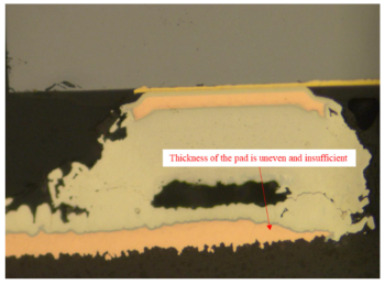
12	Multilayer construction	Skin aging	(1) The glass-fiber-reinforced composite matrix is prone to chain splitting or cross-linking during high-temperature thermal aging. (2) The internal structure of the glass-fiber-reinforced composite changes, and the chalking especially leads to yellow discoloration.	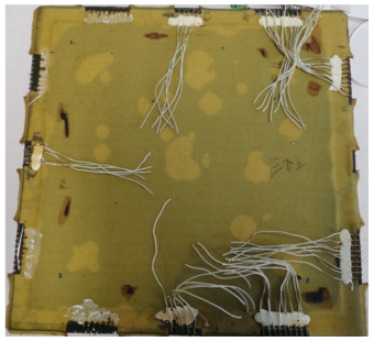
13		Skin debonding	(1) At high temperatures, the adhesive film gradually ages, the viscosity decreases, and the binding force on the skin decreases. (2) The elastic deformation and plastic deformation ability of the skin decreased significantly under high-temperature conditions. (3) Under high-temperature conditions, the thermal expansion coefficient of the skin, the film, and the PCB board differ, resulting in a greater degree of internal stress.	
14		Bubbles are detected in the honeycomb structure	Bubbles introduced during the preparation process promote failure.	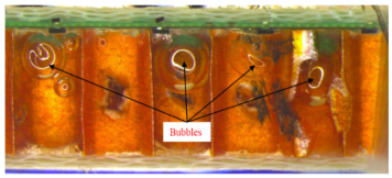
15		Bubbles are detected in the adhesive film	Bubbles that form during the preparation process can lead to failure.	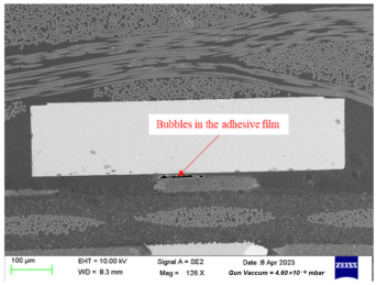

## Data Availability

Data are contained within the article.

## Abbreviations

The following abbreviations are used in this manuscript:

AFSS	Active frequency-selective surface
MEMS	Microelectromechanical system
FSS	Frequency-selective surface
FMEA	Fault mode impact and hazard analysis
FA	Failure analysis
HALT	Highly accelerated life testing
RET	Reliability enhancement testing
PCBA	Printed circuit board assembly
PCB	Printed circuit board
